# Internal modulation of proteolysis in vascular extracellular matrix remodeling: role of ADAM metallopeptidase with thrombospondin type 1 motif 5 in the development of intracranial aneurysm rupture

**DOI:** 10.18632/aging.202948

**Published:** 2021-05-02

**Authors:** Weihan Wang, Hao Zhang, Changkai Hou, Quanlei Liu, Shuyuan Yang, Zhen Zhang, Weidong Yang, Xinyu Yang

**Affiliations:** 1 Department of Neurosurgery, Tianjin Medical University General Hospital, Tianjin, China; 2 Tianjin Neurological Institute, Key Laboratory of Post-Neuroinjury Neuro-Repair and Regeneration in Central Nervous System, Ministry of Education and Tianjin City, Tianjin Medical University General Hospital, Tianjin, China; 3 Department of Neuro-Oncology and Neurosurgery, Tianjin Medical University Cancer Institute and Hospital, National Clinical Research Center for Cancer, Key Laboratory of Cancer Prevention and Therapy, Tianjin’s Clinical Research Center for Cancer, Tianjin, China

**Keywords:** intracranial aneurysm, extracellular matrix, ADAMTS-5, animal model

## Abstract

Intracranial aneurysms (IAs) are common cerebrovascular diseases that carry a high mortality rate, and the mechanisms that contribute to IA formation and rupture have not been elucidated. ADAMTS-5 (ADAM Metallopeptidase with Thrombospondin Type 1 Motif 5) is a secreted proteinase involved in matrix degradation and ECM (extracellular matrix) remodeling processes, and we hypothesized that the dysregulation of ADAMTS-5 could play a role in the pathophysiology of IA. Immunofluorescence revealed that the ADAMTS-5 levels were decreased in human and murine IA samples. The administration of recombinant protein ADAMTS-5 significantly reduced the incidence of aneurysm rupture in the experimental model of IA. IA artery tissue was collected and utilized for histology, immunostaining, and specific gene expression analysis. Additionally, the IA arteries in ADAMTS-5-administered mice showed reduced elastic fiber destruction, proteoglycan accumulation, macrophage infiltration, inflammatory response, and apoptosis. To further verify the role of ADAMTS-5 in cerebral vessels, a specific ADAMTS-5 inhibitor was used on another model animal, zebrafish, and intracranial hemorrhage was observed in zebrafish embryos. In conclusion, our findings indicate that ADAMTS-5 is downregulated in human IA, and compensatory ADAMTS-5 administration inhibits IA development and rupture with potentially important implications for treating this cerebrovascular disease.

## INTRODUCTION

Intracranial aneurysms (IAs) are common cerebrovascular diseases, and approximately 1% to 5% of the general population may harbor intracranial aneurysms [1, 2]. IA causes no symptom and goes unnoticed in most cases. Nevertheless, a ruptured IA causes subarachnoid hemorrhage that leads to high mortality and disability rates [3]. IA lesions are histologically characterized by chronic inflammation and degenerative changes in the vessel wall [4]. Additionally, extracellular matrix (ECM) degradation and remodeling is a hallmark of aneurysm formation; however, the molecular mechanisms underlying the degradation of ECM components of the arterial wall are not completely elucidated.

Metalloproteinases, which play pivotal roles in cardiovascular remodeling by degrading matrix substrates, such as MMPs (matrix metalloproteinases), contribute to arterial wall degeneration in the aneurysm pathogenetic process [5, 6]; however, they are not the only factors that are implicated in this process. Recently, a sort of novel metalloproteases, known as the ADAMTS (A disintegrin and metalloprotease with thrombospondin motifs) family, have been explored in vascular ECM. ADAMTSs are soluble proteinases involved in multiple vascular biological functions, including the degradation of ECM proteoglycans, collagen processing, and angiogenesis [7]. Thereinto, ADAMTS-5 activities have been identified that are involved in thoracic aortic aneurysm (TAA) formation and development [8]. In contrast to the extensive investigations of other metalloproteinases in aneurysmal pathogenesis, little is known about the role of ADAMTS-5 in IA formation and development.

In the present study, we characterized the regional changes in ADAMTS-5 expression in human IA wall and experimental murine IA samples. We hypothesized that ADAMTS-5 is important in the development of IA and examined this phenomenon in the angiotensin II (Ang II)-infused and elastase-induced IA mouse model, and further validated it in another model animal, zebrafish.

## RESULTS

### ADAMTS-5 is decreased in the arterial wall of aneurysmal cerebral arteries

**Figure 1:**
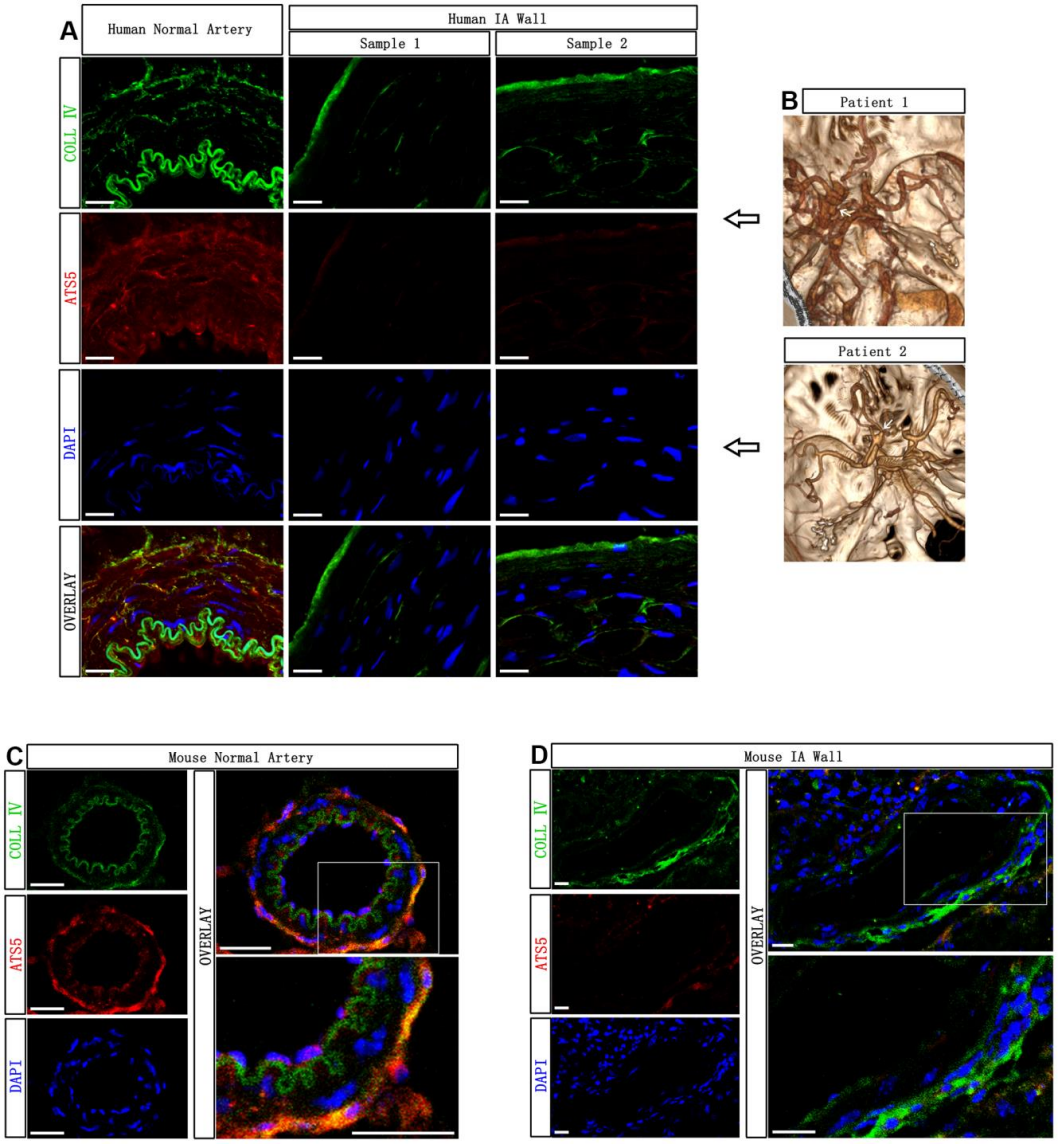
**Decreased presence of ADAMTS-5 within the aneurysmal wall.** (**A**) Representative immunofluorescence performed on intracranial aneurysm samples harvested from human IA patients after microsurgical clipping, and the normal cerebral artery harvested from a human brain trauma patient, showing the presence of ADAMTS-5. ADAMTS-5 and Collagen IV are displayed in red and green, respectively. Scale bars: 20 μm. (**B**) CTA identification of aneurysms from the patients included in this study. Human IA sample 1 and sample 2 were obtained from IA patient 1 and patient 2, respectively. (**C**, **D**) Representative immunofluorescence performed on mouse samples displaying the healthy control artery and aneurysmal artery for ADAMTS-5. Scale bars: 20 μm. ATS5, ADAMTS-5; COLL IV, Collagen IV.

First, we investigated the presence of ADAMTS-5 in the aneurysmal arteries of human tissue and compared them with normal cerebral arteries by immunostaining. The CTA imaging data of the two patients who provided human IA samples in the study are shown in Figure 1B. Human IA sample 1 and sample 2 were obtained from IA patient 1 and patient 2, respectively. Extensive ADAMTS-5-positive immunostaining was revealed in the major parts of the media and intima/adventitia for normal cerebral arteries, with only slight ADAMTS-5-positive staining in the luminal part of the intima of IA arteries (Figure 1A). To further verify the decrease in ADAMTS-5 in IA arteries, IA samples obtained from mouse models were used. Immunostainings performed from IA mice samples also revealed the reduction of ADAMTS-5 within the arterial wall of IA arteries (Figure 1C, 1D).

### Administration of rADAMTS-5 protects against IA rupture

**Figure 2:**
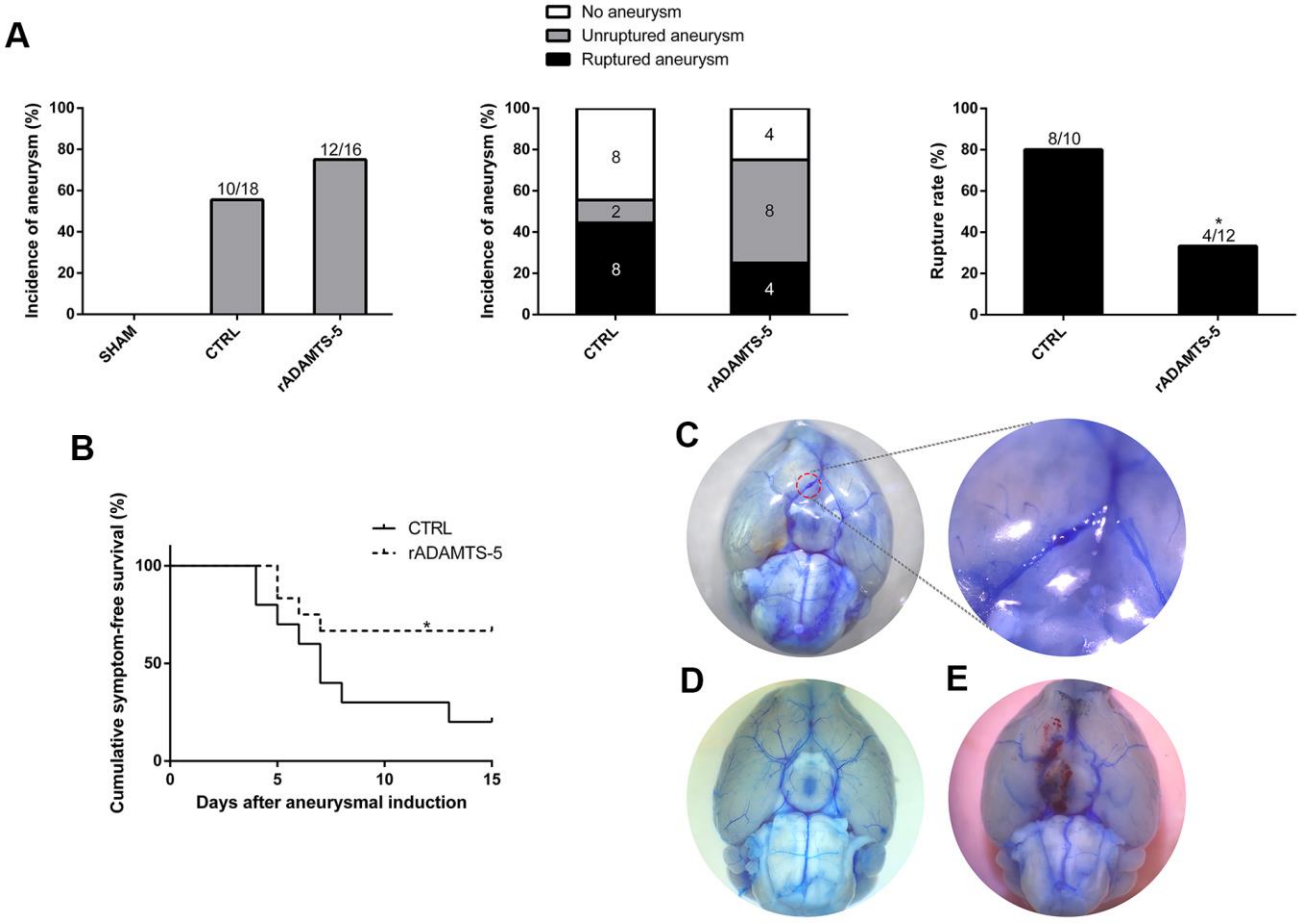
**Effects of recombinant protein ADAMTS-5 on the development of aneurysmal rupture in male mice.** (**A**) Incidence of aneurysm and rupture rate in sham mice, control (CTRL) IA mice, and recombinant protein ADAMTS-5-administered (rADAMTS-5) IA mice. (**B**) Symptom-free curve (Kaplan-Meier analysis curve) demonstrated a significant increase in survival in the rADAMTS-5 treatment cohort (log-rank *P*<0.05). (**C**) Unruptured aneurysm. (**D**) No aneurysm. (**E**) Ruptured aneurysm. *P<0.05, **P<0.01.

Given the obvious reduction in the cerebral arterial ADAMTS-5 expression observed in human IA and mouse IA, we sought to determine whether ADAMTS-5 played a protective role against IA development using the approach that giving a consecutive number of days of intraperitoneal injections of recombinant ADAMTS-5 protein (rADAMTS-5) in mice that received IA induction. Until the end of the 15-day experiment, no significant difference was found in the overall incidence of aneurysms between the control (CTRL) and rADAMTS-5 treatment group (55.56% versus 75%; n=18 versus n=16; Figure 2A). Nevertheless, rADAMTS-5 treatment significantly reduced the aneurysm rupture rate (Figure 2A; control versus rADAMTS-5: 80% versus 33.33%; *P*<0.05). The mouse group treated with rADAMTS-5 showed significantly better symptom-free survival than the control group (Figure 2B; Kaplan-Meier log-rank *P*<0.05). Figure 2D shows normal cerebral arteries, Figure 2C shows an unruptured aneurysm from a mouse without symptomatic signs throughout the experimental period, and Figure 2E shows a ruptured aneurysm with subarachnoid hemorrhage from a mouse that exhibited symptoms of aneurysmal rupture at day 7 after aneurysm induction. Additionally, the blood pressure was monitored before aneurysm induction surgery and every 5 days until day 15 of the study. No significant difference was found in the blood pressure between the control and treatment groups at any time point (Supplementary Figure 2 in the Supplementary Materials). The animal experimental procedure is shown in Supplementary Figure 1 (Supplementary Materials).

### rADAMTS-5-administered IA mice exhibit reduced cerebral arterial matrix degradation

**Figure 3:**
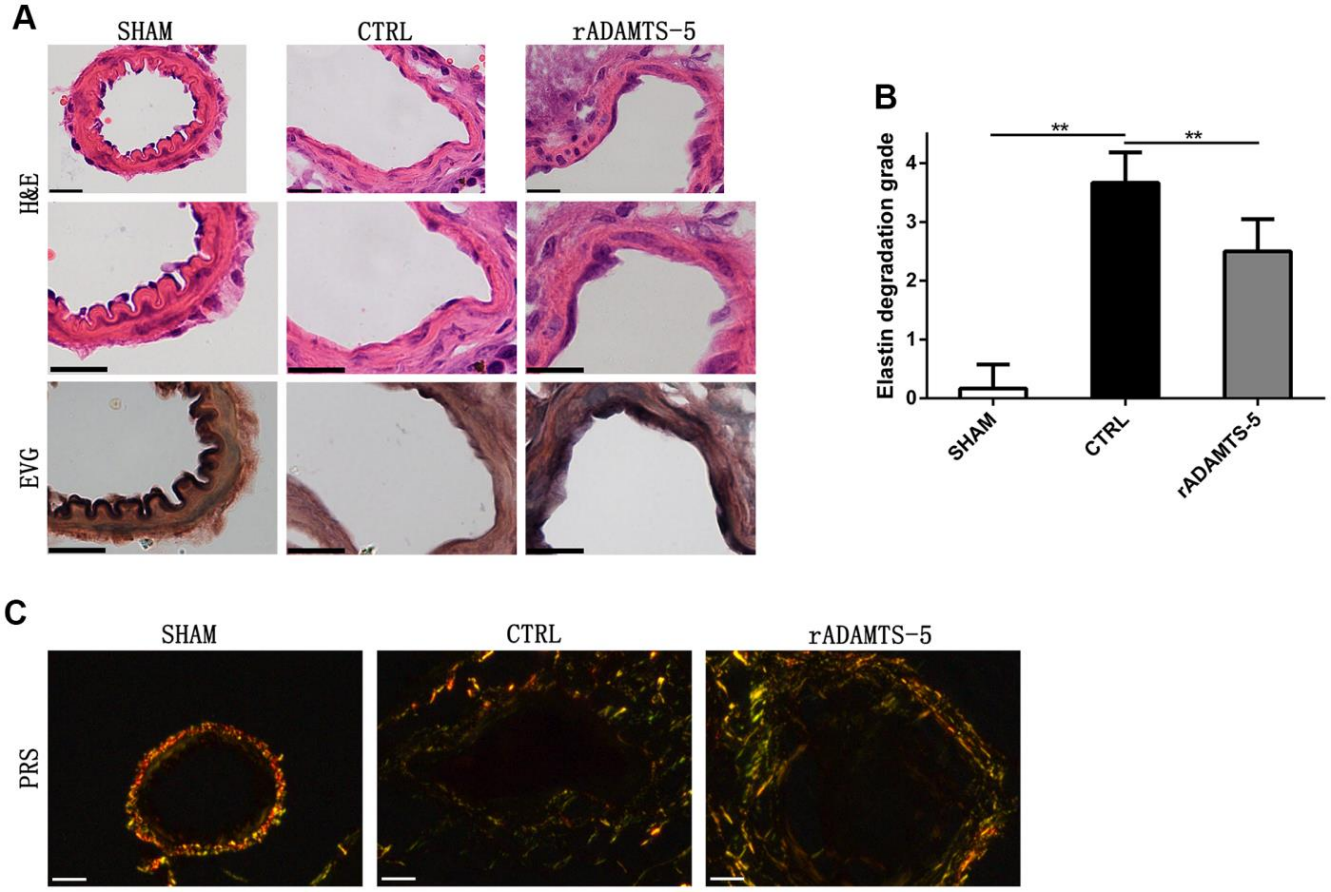
**Recombinant protein ADAMTS-5 protected elastase-induced IA mice from vascular matrix degradation.** (**A**) Representative hematoxylin and eosin (H&E) and elastin Verhoeff-Van Gieson (EVG)-stained section of cerebral arterial sections of sham mice, control (CTRL) IA mice, and recombinant protein ADAMTS-5-administered (rADAMTS-5) IA mice. Scale bar: 20 μm. (**B**) Quantification histogram showing elastin filament degradation. Arterial wall elastin filament degradation was evaluated based on the degree of degradation in elastin fibers (graded 0–4) as described in the Materials and Methods. **P<0.01. (**C**) Picrosirius red staining for collagen content within the arteries captured by polarization microscopy imaging from the sham mice, control IA mice, and recombinant protein ADAMTS-5-administered (rADAMTS-5) IA mice. Compared with the control group, the rADAMTS-5-administered group showed increased collagen birefringence under polarization. Scale bar: 20 μm.

After determining of the protective effect of rADAMTS-5 administration in the IA mouse model, we examined the role of ADAMTS-5 in protecting against arterial ECM degradation. As expected, hematoxylin and eosin staining showed conspicuous thickening of the arterial wall with massive matrix degradation and remodeling in elastase-induced IA mice (Figure 3A). Mice administered rADAMTS-5 showed reduced adventitial atrophy and less lamellar fragmentation than control IA mice (Figure 3A). Next, elastin Verhoeff-van Gieson (EVG) staining was performed to characterize the elastin integrity of IA arteries. The staining demonstrated extensive elastin degradation within the aneurysmal wall of elastase-induced IA mice compared with intact elastin found in sham mice. However, the group of mice that received rADAMTS-5 exhibited a decreased tendency of elastin degradation (Figure 3A). Semiquantitative measurements of elastin degradation showed significantly reduced elastin degradation in rADAMTS-5-administered mice compared with control IA mice (Figure 3B). To further assess collagen distribution in IA, we also performed picrosirius red staining (PRS) for collagen within the aneurysmal tissue by polarization microscopy. Polarized light for picrosirius staining analysis confirmed low mural collagen existence localized within the adventitial layer of the aneurysm section acquired from elastase-induced control IA mice. The overall picrosirius red staining area was lower in the control IA arterial wall than in rADAMTS-5-administered mice (Figure 3C).

### Macrophage infiltration and gene expression in the cerebral arteries of rADAMTS-5-administered mice

**Figure 4:**
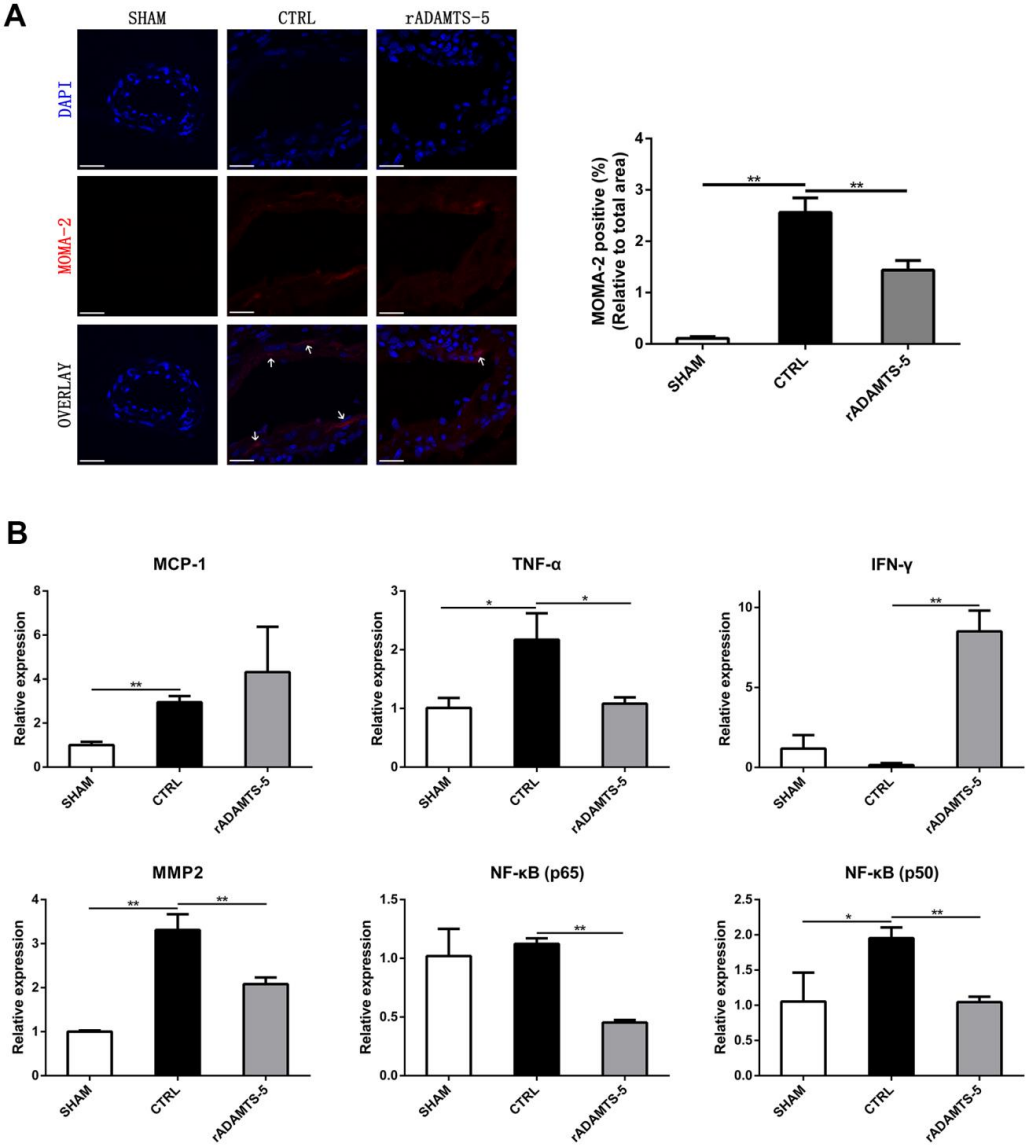
**Effects of rADAMTS-5 on monocyte/macrophage infiltration and inflammatory mediators within the IA artery.** (**A**) Immunostaining for macrophages (white arrow) with MOMA-2 (red) of arterial sections of the sham group, control group (CTRL), and rADAMTS-5-administered group. Scale bar, 40μm. (**B**) Effects of rADAMTS-5 treatment on the mRNA levels of inflammatory mediators. *P<0.05, **P<0.01.

Infiltrating macrophages in the IA wall and alterations in inflammatory mediators are associated with the formation and rupture of aneurysms. And macrophage infiltration is a common phenomenon that exists in aneurysm formation. Thus, the localization of macrophages in mouse IA arterial walls was identified by immunostaining. Sections of aneurysmal arteries of control IA mice showed a significantly higher staining area for macrophages (monocyte and macrophage antibody staining area) than those of mice receiving rADAMTS-5 during the 2 weeks of IA induction (Figure 4A). Thereafter, we assessed the role of ADAMTS-5 in the regulation of specific inflammatory mediators during IA formation and rupture. After aneurysm induction, rADAMTS-5-administered mice showed a significant difference in the expression of multiple inflammatory mediators (tumor necrosis factor-α [TNF-α], matrix metalloproteinase [MMP]-2, interferon-γ [IFN-γ], nuclear factor kappa B [NF-κB]-p65, and nuclear factor kappa B [NF-κB]-p50) when compared with control IA mice (Figure 4B). By contrast, no significant increase was found in the expression of genes encoding the mediator of monocyte chemoattractant protein-1 (MCP-1) (Figure 4B).

### rADAMTS-5 administration in IA mice reduces cerebral arterial apoptosis

**Figure 5:**
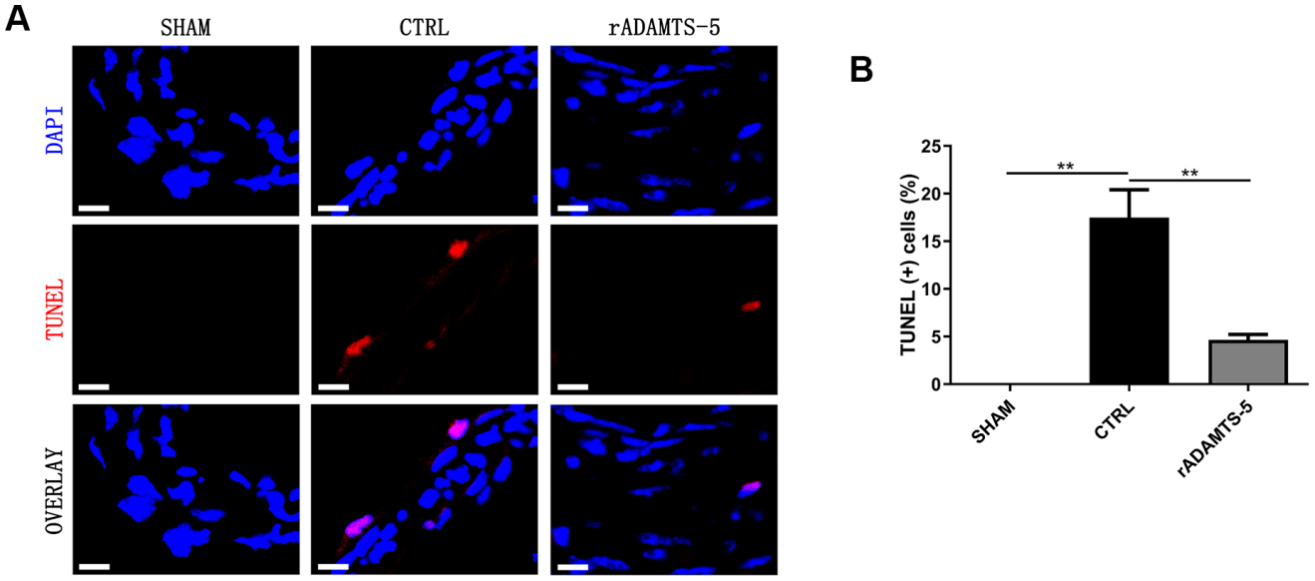
**Decreased apoptosis in the IA arteries of rADAMTS-5-administered mice.** (**A**) Representative images of TUNEL staining indicating less apoptosis (displayed in red) in arteries from rADAMTS-5-administered mice than in those from control (CTRL) mice. (**B**) Quantification of the TUNEL (+) cells in arteries from the control (CTRL) mice and rADAMTS-5-administered mice. **P<0.01.

Cell apoptosis of the arterial wall is a significant feature of aneurysmal disease and may contribute to arterial destruction and disease development [9, 10]. Therefore, we examined the role of ADAMTS-5 in arterial cell apoptosis. The *in vivo* TUNEL assay was performed to evaluate cell apoptosis in the aneurysmal wall. TUNEL-positive cells were abundant in the arterial wall of control IA mice but were significantly reduced in the arterial wall of rADAMTS-5-administered IA mice (Figure 5A, 5B). The finding suggests the potential involvement of ADAMTS-5 in apoptosis during IA development and rupture.

### ADAMTS-5-related proteolysis in the cerebral arteries of IA mice

**Figure 6:**
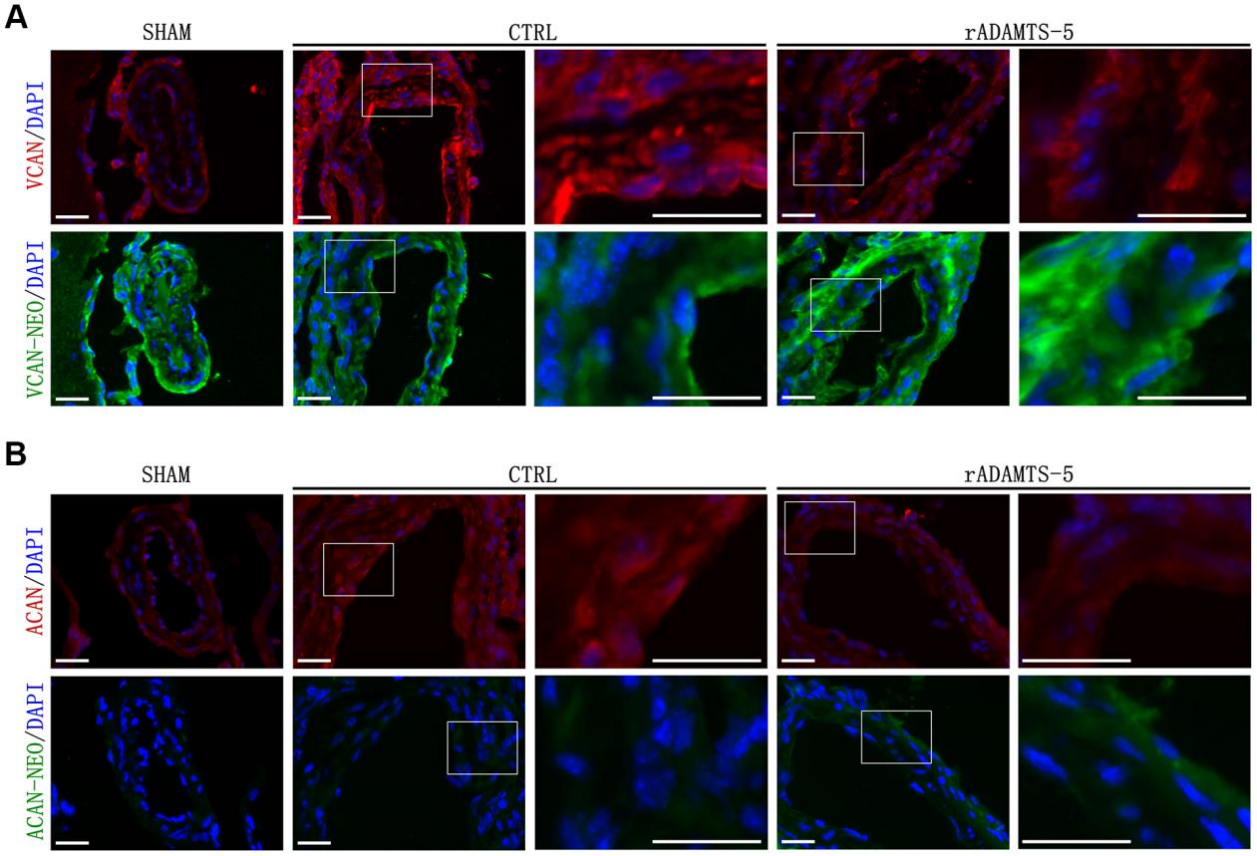
**Effects of rADAMTS-5 on proteolysis and detection of the main proteoglycans in IA arteries.** Representative immunofluorescence staining images showing (**A**) versican (VCAN) degradation and (**B**) aggrecan (ACAN) degradation in IA arteries from sham mice and control (CTRL) mice and in arteries from rADAMTS-5-administered mice. Images in columns 3 and 5 represent higher magnification views of the areas contained within the white boxes in the left-hand columns. Versican and aggrecan are displayed in red, and the neoepitopes of versican and aggrecan are displayed in green. Scale bar: 25 μm.

Members of the ADAMTS family are the enzymes responsible for aggregated proteoglycan cleavage [11–13]. Versican and aggrecan can be cleaved by different proteases including MMPs and several members of the ADAMTS family [14, 15]. Immunofluorescence staining revealed the increased presence of versican in the aneurysmal wall of control IA mice compared with that in the healthy arterial wall of sham mice. Additionally, an increased tendency was observed in the cleavage products of versican (neoepitope of versican) in the aneurysmal wall of rADAMTS-5-administered IA mice compared with that in the arteries of control IA mice (Figure 6A), indicating the involvement of ADAMTS-5 in versican degradation during IA formation. Furthermore, aggrecan, another large aggregating proteoglycan, was also accumulated in the aneurysmal artery wall after IA induction in mice. We also found that the administration of rADAMTS-5 in IA mice reduced the aberrant accumulation of aggrecan. This reduction was accompanied by an opposite increase in the degradation products of the proteoglycan aggrecan (neoepitope of aggrecan) (Figure 6B).

### Validation of the role of ADAMTS-5 in cerebral vessels in the zebrafish animal model

**Figure 7:**
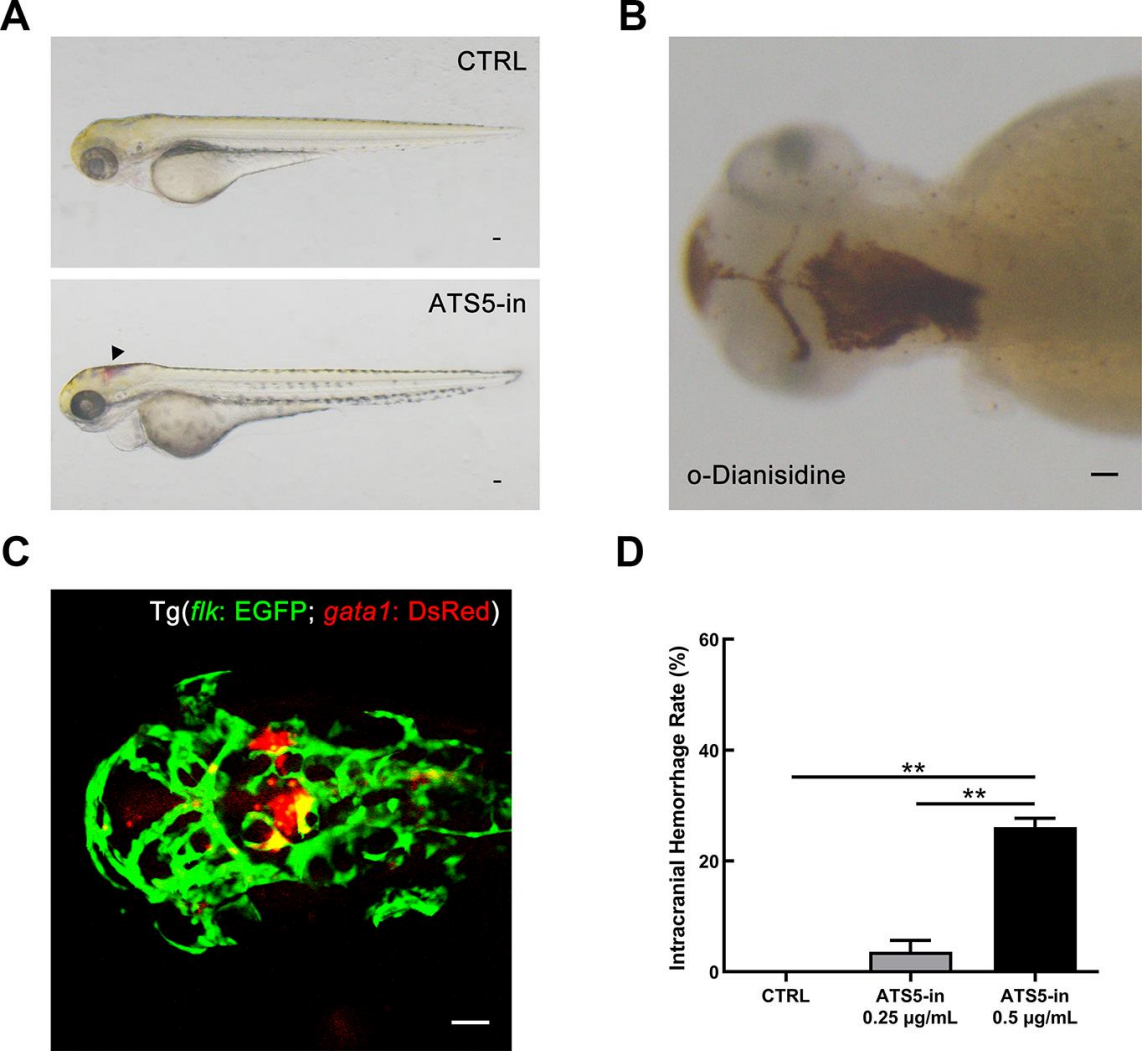
**Inhibition of ADAMTS-5 causes intracranial hemorrhage in zebrafish.** (**A**) Zebrafish embryos treated with DMSO (control, CTRL group), and intracranial hemorrhage in embryos treated with 0.5 μg/mL of ADAMTS-5 inhibitor (ATS5-in). The black arrow indicates the bleeding site. (**B**) O-Dianisidine staining confirming intracranial hemorrhage at 72 hpf. (**C**) Confocal microscopy showing cerebral blood vessel leakage of the Tg (*flk*: EGFP; *gata1*: DsRed) embryo. (**D**) Incidence rates of intracranial hemorrhage of the control group (CTRL) and ADAMTS-5 inhibitor treatment group (ATS5-in) at 0.25 μg/mL and 0.5 μg/mL. Scale bar: 50 μm. **P<0.01.

ADAMTS-5 inhibitor (Supplementary Figure 3 in Supplementary Materials) treatment was performed in zebrafish embryos. Compared with the fish embryos of the control group (exposed to 0.1% DMSO, v/v), which exhibit normal development, intracranial hemorrhage was observed in the group treated with ADAMTS-5 inhibitor (ATS5-in 0.5 μg/mL) from 36 hpf to 72 hpf (Figure 7A). O-Dianisidine staining at 72 hpf confirmed the presence of intracranial hemorrhage in those embryos (Figure 7B). We further determined the effect of ADAMTS-5 inhibitor using the Tg (*flk*: EGFP; *gata1*: DsRed) transgenic zebrafish line, in which vascular endothelial cells are labeled with EGFP (enhanced green fluorescent protein), and red blood cells are labeled with DsRed (*Discosoma* sp. red fluorescent protein). The results of confocal microscopy showed that intracranial hemorrhage occurred in the midbrain and hindbrain of the fish embryos (Figure 7C). According to the statistical data, the rate of intracranial hemorrhage was significantly higher in the high-dose ADAMTS-5 inhibitor treatment group than in the low-dose group and DMSO-treatment control group (Figure 7D). The results demonstrated that ADAMTS-5 inhibition may weaken the integrity of the brain blood vessels, leading to intracranial hemorrhage.

## DISCUSSION

In the current study, we provide evidence that the abnormal absence of ADAMTS-5 plays an important role in arterial destruction and IA development. We found decreased expression of ADAMTS-5 in aneurysmal tissue samples from patients with IA and rADAMTS-5 administration in IA mice reduced arterial matrix degeneration and aneurysm rupture in the cerebral artery using an IA mouse model. The administration also reduced inflammatory cell infiltration and arterial cell apoptosis and led to attenuated proteolysis (proteoglycan degradation) improvement. Additionally, experiments performed on zebrafish animal model demonstrated that functional inhibition of ADAMTS5 in vascular tissue might weaken the integrity of the cerebral vessel wall, leading to intracranial hemorrhage. Taken together, these results suggest that ADAMTS-5 maladjustment is a noteworthy component in the development of IA.

The ADAMTS enzyme family comprises 19 secreted, extracellular enzymes that contain thrombospondin type 1 sequence repeat motifs common to extracellular matrix proteins and have been explored in inflammatory diseases and tissue destruction in cancer metastasis [16–19]. The associations between IA susceptibility and genetic variations in members of the ADAMTS family have been reported previously [20–22], resulting in this gene family tending to be a potential target for IA mechanistic studies and genetic screening in this disease. A few studies have illustrated aberrant regulation of ADAMTS-5 in vascular diseases, including atherosclerosis [23, 24], cerebral cavernous malformation [25], calcific aortic valve disease (CAVD) [26], and aortic dilatation [27]. Thereafter, data of the present study demonstrated that the presence of ADAMTS-5 is significantly decreased in the arterial wall of human IA and mouse IA. Thus, the typical loss of ADAMTS-5 in the aneurysmal wall may be involved in arterial destruction through several mechanisms.

Excessive accumulation of proteoglycan, elastic fiber fragmentation, and abnormal SMC loss are defining components of vascular medial degeneration [28]. In vascular ECM, proteoglycans such as versican, which was identified in vascular tissue or synthesized by vascular cells, also increases in vascular injury/lesions [29, 30]. Therefore, aneurysmal genesis and propagation may also be accompanied by increased synthesis of proteoglycans, and overabundant proteoglycans cannot be easily degraded, contributing to the aggravation of this vascular disease. In this process, the role of ADAMTS protease is particularly important. The expression levels of these proteases, such as ADAMTS-5, are reduced, as we confirmed in the present study, they play a role in promoting the abnormal accumulation of proteoglycans. A subdued degradation of proteoglycans caused by reduced ADAMTS-5 may be an underlying mechanism for its contribution to vascular diseases. In this study, the reduction of proteoglycans degradation in the IA wall was observed, and versican/aggrecan accumulation was correlated with decreased ADAMTS-5 expression levels. The recovered versican/aggrecan degradation products in rADAMTS-5-administered mice suggest that ADAMTS-5-mediated versican/aggrecan degradation may be partially responsible for arterial destruction and IA development. The present results demonstrate that rADAMTS-5 does not have a significant effect on the incidence rate of aneurysms. Intracranial aneurysm is a progressive disease. At the initial stage of the disease, pathological vascular remodeling has not quite begun, and the extent of the vascular lesion is relatively slight. At this time, ADAMTS-mediated proteolysis for proteoglycan regulation is at a normal level. With aneurysm growth and development, an increasing number of proteoglycans accumulate in the vascular wall, and ECM remodeling has started throughout the vessel wall, leading to continuous weakening of the blood vessel. However, ADAMTS-5 could play a better role in the later stage of pathological progression, and rADAMTS-5 was shown to have a significant impact on the rupture rate of aneurysms.

Proteoglycan binds to hyaluronan and interacts with link protein to form aggregates [31–33], which localize in the ECM space in addition to fibrous proteins such as collagen and elastic fibers. Normal physiological levels of interstitial proteoglycans produce optimal osmotic expansion pressure, enabling the elastic lamellar to withstand cyclic compression [34–36]. However, excess swelling pressure generated by aggrecan or versican, which may contribute to cellular dysfunction, may be a major factor for SMC deterioration and maladaptive behavior in the vessel wall, even leading to cell apoptosis [32, 36, 37]. Additionally, the abnormal interstitial swelling pressure generated from the pathologic accumulation of proteoglycans may mechanically disrupt fibrillar ECM, leading to elastic fiber fragmentation and involvement in IA initiation and development [32, 38]. Our present data showed fewer elastin filament breaks and fewer apoptotic cells in the IA artery from rADAMTS-5-administered IA mice than in the artery from control IA mice. Therefore, we propose that the generation of versican/aggrecan accumulation in the IA wall by ADAMTS-5 loss contributes to rupture risk, and this pathologic process includes on-going degradation of the internal elastic lamina and arterial cell death in the IA wall.

Versican can bind to hyaluronan as well as CD44 [39], indicating that versican may be involved in the stabilization of CD44-dependent interactions in inflammatory cells. Additionally, versican and hyaluronan facilitate the adhesion of monocytes/macrophages [40]; versican can interact with inflammatory chemokines and partly regulates the activity of these factors [31, 41]. Our findings suggest that ADAMTS-5 may retard IA development and rupture by suppressing arterial inflammation; the reduced inflammatory infiltration is likely due to less versican accumulation in the aneurysmal wall caused by the compensatory increase in ADAMTS-5. Similarly, aggrecan, another proteoglycan that large aggregates in cartilage [42], was observed to accumulate in vessels after IA induction. Aggrecan accumulation was reported to be induced after vascular injury, and it was accompanied by reduced expression of ADAMTS-1 and ADAMTS-5 [43]. Our data also support that the reduction of aggrecan cleavage correlates with the absence of ADAMTS-5 in the aneurysmal wall. Additionally, proteoglycans, including aggrecan and versican, present growth factors and cytokines to the surrounding tissue, and the interactions between them and ambient ECM components could regulate multiple cellular responses, including inflammation [44, 45]. A balance between proteoglycans and their ADAMTS-cleaved fragments may influence the progression of ECM remodeling in the IA wall via inflammatory pathways. However, further studies are needed to explore whether aggrecan/versican or their cleavage products may directly contribute to the inflammatory process during the onset of IA.

The zebrafish has proven useful to visualize intracranial hemorrhage, making it possible to observe compound-treated animals for hemorrhage defects conveniently [46]. In order to further verify the functions and role of ADAMTS-5 in cerebral blood vessels, we used the specific ADAMTS-5 inhibitor in zebrafish embryos and observed intracranial hemorrhage in the midbrain and hindbrain of the larvae. Thus, the prominent phenotypes of intracranial hemorrhage were caused by ADAMTS-5 deficiency, revealing that the absence of ADAMTS-5 may lead to specific defects in the maintenance of vascular integrity. This finding also validates the important role of ADAMTS-5 in maintaining structural balance and physiological homeostasis in the vessel wall.

However, the present study has limitations. A mouse model of elastase injection was used to study the effects of ADAMTS-5 on IA that may not recapitulate all aspects of a human intracranial aneurysm, particularly regarding actual hemodynamics and mechanical properties in the cerebral vessel [47, 48]. Additionally, the relative importance of different members of the ADAMTS family may differ between species.

Overall, this study indicates that ADAMTS-5 plays an important role in IA development. The absence of ADAMTS-5 in IA arteries attenuates proteoglycan degradation and leads to the accumulation of proteoglycans (versican/aggrecan), along with elastic fiber fragmentation and collagen content impairment, which may disrupt ECM integrity, facilitate ECM destruction and remodeling, and induce inflammation and apoptosis in the cerebral artery, potentially contributing to the pathogenesis of IA. Based on these findings, it is expected that further research will help us better understand the ADAMTS-5-mediated regulation of proteoglycan dynamics, which may provide new targets for nonsurgical IA therapy.

## MATERIALS AND METHODS

### Animal study design

Experiments or animal surgery procedures were conducted in accordance with the guidelines approved by the Tianjin Medical University General Hospital, Institutional Animal Care and Use Committee.

### Mouse model study

IAs were induced in mice as previously described [49, 50], all in a C57BL/6 background and 8~10 weeks old. Briefly, a single injection of elastase (35 mU in 2.5 μL; Sigma-Aldrich, St. Louis, MO, USA) into the cerebrospinal fluid at the right basal cistern was realized in anesthetized animals, followed by subcutaneous implantation of an osmotic pump (Durect Corporation, Cupertino, CA, USA) filled with angiotensin II (1000 ng/kg/min; Sigma-Aldrich) to induce systemic hypertension. Both the vehicle control group (CTRL group) and the rADAMTS-5 group received elastase injection and angiotensin II infusion, the sham group received sterile saline. Systolic blood pressure was assessed by repeated measurements using the tail-cuff method. To detect IA rupture, neurological examination was performed daily by 2 blinded observers, as previously described [50, 51]. Animals were analyzed twice a day for the detection of neurological symptoms during 15 days postinduction. Neurological symptoms were scored as follows: 0, normal function; 1, reduced eating or drinking activity demonstrated by a weight loss >2.0 g of body mass (≈10% weight loss) over 24 hours; 2, flexion of the torso and forelimbs on lifting the whole animal by the tail; 3, circling to one side with a normal posture at rest; 4, leaning to one side at rest; and 5, no spontaneous activity. Symptomatic mice (neurological symptom score, 1-5) were immediately euthanized (within 3 hours) to identify intracranial hemorrhage (ruptured aneurysms). Before symptoms occur, the mice were considered as symptom-free surviving animals, and all asymptomatic mice were euthanized 15 days after aneurysm induction. The brain samples were perfused with phosphate-buffered saline followed by gelatin containing blue dye to visualize cerebral arteries. Aneurysms are defined as a localized outward bulging of the vascular wall whose diameter is greater than the parent artery diameter [49, 52].

We started the administration of recombinant protein ADAMTS-5 (R&D SYSTEMS, Minneapolis, MN, USA) 1 day before aneurysm induction and continued the treatment for 2 weeks. Eighteen mice were administered rADAMTS-5 at a dosage of 7.5 μg/kg/day through intraperitoneal injection (200 μL injection volume), and sixteen mice in the vehicle control group received a phosphate-buffered saline injection.

### Zebrafish husbandry and chemical treatments

Zebrafish (Danio rerio) husbandry and embryo maintenance were performed under standard laboratory conditions according to the Institutional Animal Care and Use Committee protocols. The AB wild-type strain and transgenic line Tg (*flk*: EGFP; *gata1*: DsRed; endothelial cells are marked by EGFP, and erythrocytes are indicated by DsRed) were used in this study. For chemical treatment, the embryos were incubated from 5 to 72 hpf in E3 egg water supplemented with ADAMTS-5 inhibitor (APExBIO, Houston, TX, USA) or DMSO (dimethyl sulfoxide, Sigma-Aldrich). The embryos were treated with 0.003% 1-phenyl-2-thiourea (PTU, Sigma-Aldrich) to avoid pigmentation. Thirty embryos were incubated in each group.

### O-Dianisidine staining

Hemoglobin leakage was detected at 3 dpf by performing o-dianisidine staining as previously described [53]. Dechorionated embryos were collected and stained for 15 min in the dark with 0.6 mg/mL of o-dianisidine (Sigma-Aldrich), 0.01 M sodium acetate (pH 4.5), 0.65% H_2_O_2_ and 40% (v/v) ethanol at room temperature, followed by three washes with PBS. The stained embryos were immediately analyzed by microscopy (Olympus, Tokyo, Japan).

### Immunostaining

### Human samples

Human aneurysm samples were obtained during the surgeries by resecting the aneurysm sac distal to the clip closing the neck. Samples were obtained from two patients: a 69-year-old woman who had subarachnoid hemorrhage after a rupture of the posterior communicating artery IA (patient 1); a 40-year-old man who had subarachnoid hemorrhage after a rupture of the anterior communicating artery IA (patient 2). Normal cerebral vascular specimens were acquired from emergency surgery for brain trauma. The tissues were immediately stored for 2 hours in a fixative and then were cryoprotected before freezing in Tissue-Tek (Sakura, Tokyo, Japan). Experiments with the clinical samples were performed according to the principles expressed in the Declaration of Helsinki, and informed consent was obtained.

### Mouse tissue samples

Each mouse was anesthetized and transcardially perfused with cold phosphate-buffered saline, followed by 20 mL of fixative solution (4% paraformaldehyde). The brains were cryoprotected before freezing in Tissue-Tek (Sakura).

### Staining and confocal microscopy

For both human and mouse tissue samples, cryomicrotome-cut transversal sections (10 μm) were collected on polylysine slides and stored at −80° C before staining. Briefly, mouse and human tissue sections were blocked with 5% normal goat serum containing 0.1% Triton X-100 for 60 min (room temperature). Sections were incubated overnight with the following primary antibodies: collagen IV (Southern Biotech, Birmingham, AL, USA); ADAMTS-5, MOMA-2, versican-neo (the DPEAAE neoepitope of versican, neoepitope on versican generated by ADAMTS cleavage), and aggrecan (Abcam, Cambridge, MA, USA); aggrecan-neo (the NITEGE neoepitope of aggrecan) and versican (Thermo Fisher Scientific, Waltham, MA, USA). The sections were then incubated with the appropriate secondary antibody (Alexa Fluor 488, 594, or 647, and FITC or Cy3, Abcam). The sections were washed and coverslipped with antifade medium containing DAPI (Abcam), and images were digitally captured using an Olympus FluoView 1200 confocal microscope, followed by analysis using Image J (NIH, USA) software.

For zebrafish, living larvae were embedded in 1.2% low melting agarose, which was dissolved in E3 medium and Tricaine (Sigma-Aldrich). Tg (*flk*: EGFP; *gata1*: DsRed) embryos at 48 hpf with intracranial hemorrhage were captured by confocal microscopy (Carl Zeiss, Jena, Germany).

### Gene expression in mouse cerebral arteries

Cerebral arteries (Circle of Willis, including the middle cerebral arteries and basilar artery) were isolated from the mouse brain when possible and dissolved in TRIzol (Invitrogen, Carlsbad, CA, USA). Quantitative reverse transcription-polymerase chain reaction (qRT-PCR) was used to quantify the mRNA levels. Quantitative values were obtained from the threshold cycle value (CT), and the data were analyzed by the 2^−∆∆CT^ method. We assessed the mRNA expression of monocyte chemoattractant protein-1 (MCP-1), tumor necrosis factor-α (TNF-α), interferon-γ (IFN-γ), matrix metalloproteinase-2 (MMP-2), and nuclear factor kappa B (NF-κB). The transcript amount of glyceraldehyde-3-phosphate dehydrogenase (GAPDH) was quantified as an internal RNA control.

### Histological assessments

Aneurysmal artery sections were subjected to hematoxylin and eosin staining (Solarbio, Beijing, China), Verhoeff-van Gieson elastin staining (Abcam), and picrosirius red staining (Solarbio) according to the manufacturer’s instructions. Two independent observers who were blinded to the animal group allocation examined 3-6 arterial sections from mice per group. The extent of elastin fiber integrity was scored on a scale of 0 to 4 (0, no elastin degradation; 1, mild degradation; 2, moderate; 3, moderate to severe; and 4, severe elastin degradation).

### TUNEL assay

To study apoptosis in the aneurysmal artery, we performed TUNEL assays using an *in situ* cell death detection kit (KeyGEN BioTECH, Nanjing, China) according to the manufacturer’s instructions. In brief, frozen sections of the cerebral artery were incubated with 1% of Triton X-100 in PBS for 5 min at room temperature (RT). After a wash in PBS, the sections were then incubated with 50 μL of TUNEL reaction mixture for 60 min at 37° C in a humidified atmosphere in the dark. Subsequently, the nuclei were counterstained with DAPI (4′,6-diamidino-2-phenylindole) for 10 min in the dark. After staining, the sections were observed using an Olympus FV1200 confocal microscope. For each treatment group, images from three randomly selected views were captured. For each picture, the number of positive cells and total nuclei were quantified, and the percentage of positive cells was calculated.

### Statistical analysis

All of the results were expressed in the form of means ± SD. Fisher’s exact test was used to analyze the incidence of aneurysms (number of mice with any ruptured or unruptured aneurysms/total number of mice), the incidence of ruptured aneurysms (number of mice with ruptured aneurysms/total number of mice), and rupture rate (number of mice with ruptured aneurysms/number of mice with any aneurysms). Survival analyses were performed using the Kaplan–Meier method and the log-rank test. Mice that did not develop aneurysms were excluded in the survival analysis. The mRNA levels, intracranial hemorrhage rate, and blood pressure data were analyzed using Student’s t test or two-way ANOVA. GraphPad Prism 6 (GraphPad Software Inc.) was used for statistical analysis, and a *P* value <0.05 was considered statistically significant.

## Supplementary Material

Supplementary Figures
