# Supplementary material for: Internal modulation of proteolysis in vascular extracellular matrix remodeling: role of ADAM metallopeptidase with thrombospondin type 1 motif 5 in the development of intracranial aneurysm rupture
Source: Aging (Albany NY). 2021 May 2;13(9):12800–16. doi: 10.18632/aging.202948 (PMC8148490; doi:10.18632/aging.202948)
Supplement: Supplementary Figures [file aging-13-202948-s001.pdf]

SUPPLEMENTARY FIGURES

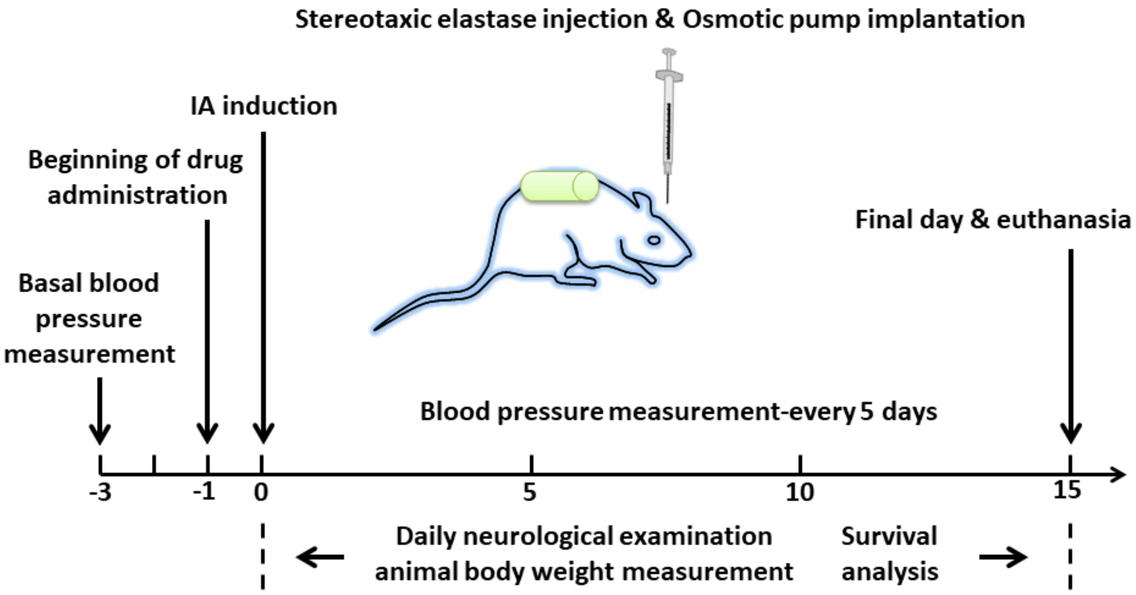

Supplementary Figure 1. Experimental protocol for IA induction. Male mice maintained with systemic hypertension induced by a chronic infusion of angiotensin II, meanwhile, also received a single stereotaxic injection of elastase to induce IAs. A neurological evaluation score during the next 15 days was used to identify symptomatic and asymptomatic IA mice.

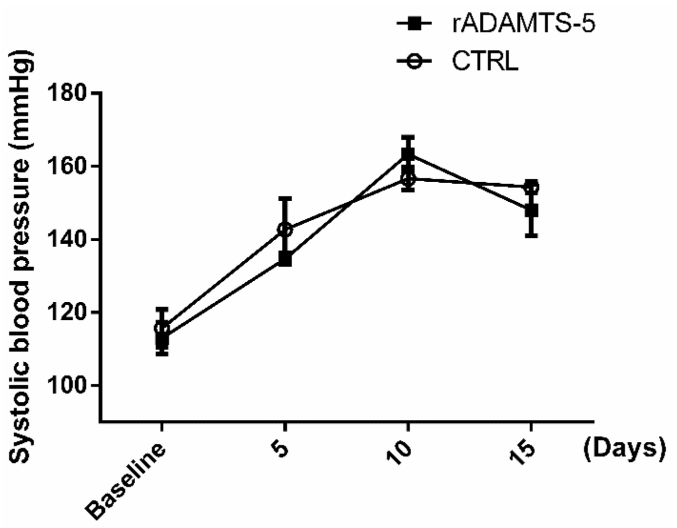

Supplementary Figure 2. Blood pressure assessment.

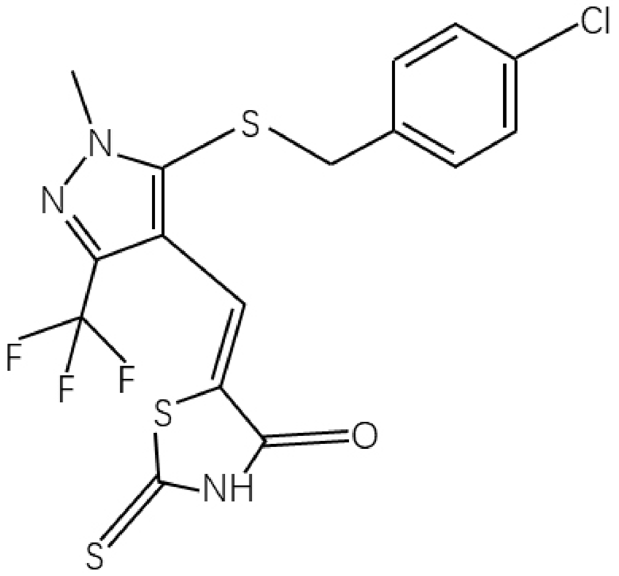

Supplementary Figure 3. The chemical structure of ADAMTS-5 inhibitor. Chemical name: (Z)-5-((5-((4-chlorobenzyl)thio)-1-methyl-3-(trifluoromethyl)-1H-pyrazol-4-yl)methylene)-2-thioxothiazolidin-4-one.
